# Kidney Bean: A Major Sensitizer among Legumes in Asthma and Rhinitis Patients from India

**DOI:** 10.1371/journal.pone.0027193

**Published:** 2011-11-09

**Authors:** Ramkrashan Kasera, Bhanu Pratap Singh, Sakuntala Lavasa, Komerla N. Prasad, Ramesh C. Sahoo, Anand B. Singh

**Affiliations:** 1 Allergy and Immunology Section, Institute of Genomics and Integrative Biology, Delhi, India; 2 University of Pune, Ganeshkhind, Pune, India; 3 USLavasa Medical & Research Center, Chandigarh, India; 4 Bangalore Allergy Centre, Bangalore, India; 5 Manipal Medical College, Mangalore, India; French National Centre for Scientific Research, France

## Abstract

**Background:**

The prevalence of IgE mediated food allergies has increased over the last two decades. Food allergy has been reported to be fatal in highly sensitive individuals. Legumes are important food allergens but their prevalence may vary among different populations. The present study identifies sensitization to common legumes among Indian population, characterizes allergens of kidney bean and establishes its cross reactivity with other legumes.

**Methodology:**

Patients (n = 355) with history of legume allergy were skin prick tested (SPT) with 10 legumes. Specific IgE (sIgE) and total IgE were estimated in sera by enzyme-linked immunosorbent assay. Characterization of kidney bean allergens and their cross reactivity was investigated by immunobiochemical methods. Identification of major allergens of kidney bean was carried out by mass spectrometry.

**Principal Findings:**

Kidney bean exhibited sensitization in 78 (22.0%) patients followed by chickpea 65 (18.0%) and peanut 53 (15%). SPT positive patients depicted significantly elevated sIgE levels against different legumes (r = 0.85, p<0.0001). Sera from 30 kidney bean sensitive individuals exhibited basophil histamine release (16–54%) which significantly correlated with their SPT (r = 0.83, p<0.0001) and sIgE (r = 0.99, p<0.0001). Kidney bean showed eight major allergens of 58, 50, 45, 42, 40, 37, 34 and 18 kDa on immunoblot and required 67.3±2.51 ng of homologous protein for 50% IgE inhibition. Inhibition assays revealed extensive cross reactivity among kidney bean, peanut, black gram and pigeon pea. nLC-MS/MS analysis identified four allergens of kidney bean showing significant matches with known proteins namely lectin (phytohemagglutinin), phaseolin, alpha-amylase inhibitor precursor and group 3 late embryogenesis abundant protein.

**Conclusion/Significance:**

Among legumes, kidney bean followed by chick pea and peanut are the major allergic triggers in asthma and rhinitis patients in India. Kidney bean showed eight major allergens and cross reacted with other legumes. A combination of SPT, sIgE and histamine release assay is helpful in allergy diagnosis.

## Introduction

Food allergy is an important public concern and there are studies to identify and characterize certain food allergens [1]. Theoretically, all food products that contain protein may cause allergic reactions. However, it depends on genetic factors, exposure to new allergenic products early in life and dietary habits. Over the last two decades, the prevalence of food allergy has doubled and its phenotypic expression increased in Westernized societies [2], [3]. Legumes are the major source of dietary ingredients throughout the world. Thus, the chances of allergic reaction to legumes are more in the atopic population. IgE mediated food allergies are reported to be 3–4% in adults and 6% in children [4]. However, perceived adult food hypersensitivity varies (1.3–19.1%) largely across different countries [5]. Studies demonstrate that food allergies are associated with asthma and rhinitis which may lead to life threatening anaphylactic reaction [6], [7]. Studies in children also show the relationship between food allergy and asthma morbidity [8], [9]. Teenagers and young adults appear to be at higher risk for fatal food allergies. The most frequently reported allergenic foods are cow's milk, hen's egg, fish, seafood, legumes, wheat and additives [1], [10], [11]. In India, the knowledge about food hypersensitivity is limited to a few clinico-immunological studies. This shows evidence of allergy to foods such as egg, milk, chickpea, rice and black gram in children and adult population [12]–[15].

Immunoglobulin E [IgE] mediated allergic reactions to legume like lentil (*Lens culinaris*) has been reported from Mediterranean paediatric patients [16]. Raised specific IgE (sIgE) was demonstrated to crude and boiled lentil proteins in sera of lentil sensitive children [17]. Isolation and characterization of relevant allergens was also performed from boiled lentil [18]. Sensitization and severe systemic reaction to red kidney bean (*Phaseolus vulgaris*) are recorded in some cases [19], [20]. Further, studies have proven allergies to chickpea and black gram in asthma and rhinitis patients and characterized their allergens [13], [14]. India is one of the major consumers of legumes since these are an important source of dietary proteins for a large population. However, studies on allergic sensitization to these legumes are confined to few types and the diagnosis is based on various *in vitro* and *in vivo* methods which suggested double blind placebo controlled food challenge (DBPCFC) as the gold standard [4], [14], [15].

The present study was aimed to determine sensitization pattern against commonly consumed legumes in India and to find a correlation among *in vivo* (SPT) and *in vitro* (sIgE , total IgE and basophil histamine release) tests, which may provide quick and easier diagnostic tool, obviating the difficulties associated with DBPCFC. In addition, IgE binding components of kidney bean (a major sensitizer) and its cross reactivity with other legumes was investigated by immunobiochemical methods using hypersensitive patients' sera.

## Methods

### Ethics statement

The present study protocol was approved by the human ethics committee of Institute of Genomics and Integrative Biology, Delhi. Informed written consent was obtained from patients and nonallergic volunteers for participation in the study.

### Study subjects

The study included allergic rhinitis and asthma patients (n = 355) with mean age 30.7±13.9 and history of legume allergy from the two centres; Bangalore Allergy Centre, Bangalore (n = 198) and USLavasa Medical and Research Centre, Chandigarh (n = 157), India. Both the clinical centres are located in geographically distinct places in India in south and north respectively. The food habits also varied among two groups of patients. Of the total, 279 (78.5%) patients were suffering from rhinitis, 11 (3.5%) with asthma and 65 (18.0%) were suffering from both. Interestingly no case of asthma alone was diagnosed from Bangalore centre (Table 1). The diagnosis of asthma and rhinitis was ascertained following American Thoracic Society guidelines, 1991 and Allergic Rhinitis and its Impact on Asthma guidelines, 2001 [21], [22]. Patients reporting symptoms such as anaphylaxis, redness of mouth, urticaria, nausea, vomiting, diarrhea, abdominal cramps, running nose or breathlessness after ingestion of legumes were included in the study. Skin prick tests (SPT) were performed with common legume extracts (1∶10 w/v) along with a panel of inhalant allergen extracts (procured commercially) from pollen, fungi and insects [14]. The SPT reactions were observed after 20 minutes and wheal diameter ≥3 mm were considered positive and graded from 1+ to 3+ based on wheal diameter [23].

**Table 1:** Demographic details of patients skin prick tested with different allergen extracts.

	Bangalore	Chandigarh	Total
Total Patients	198	157	355
Male	100 (50.5%)	93 (59.0%)	193(54.5%)
Female	98 (49.5%)	64 (41.0%)	162(45.5%)
Mean Age	29.8±13.5	31.9±14.2	30.7±13.9
Age Group (Years)			
3 to 9 (children)	15(8%)	9 (6%)	24(7%)
>10 (adult)	183(92%)	148 (94%)	331(93%)
			
Allergic rhinitis (AR)	48(24.0%)	17(11.0%)	65(18.0%)
Bronchial asthma (BA)	0	11(7.0%)	11(3.5%)
AR + BA	150(76.0%)	129(82.0%)	279(78.5%)
**Allergen**	**% Sensitization (SPT +ve)**		
Pollen	104(52.5%)	88(56.0%)	192(54.0%)
Mite	177(89.0%)	7(4.5%)	184(52.0%)
Insect	132(66.5%)	19(12.0%)	151(42.5%)
Fungus	35(17.5%)	72(46.0%)	107(30.0%)
Foods	101(49.0%)	107(68.0%)	208(58.5%)

### Preparation of extracts

Healthy seeds of selected legumes (Table 2) were crushed and defatted in diethyl ether at 4°C. The extraction was carried out following the protocol by Kumari *et al*. [14]. Extraction of legume antigens after boiling for different time and temperature (15, 30, 45, 60 minutes at 100°C and 15 minutes at 121°C) was also done along with the raw legumes. Protein content of extracts was determined by modified Lowry's method [24].

**Table 2:** Sensitization to legume allergens identified by skin prick tests in patients of asthma, rhinitis or both.

	Bangalore		Chandigarh		Total		
Legume extract	PatientsSPT No.	Patients SPT+veNo (%)	PatientsSPT No.	Patients SPT+veNo (%)	PatientsSPT No.	Patients SPT+veNo (%)	p-value*
Kidney bean(*Phaseolus vulgaris*)	198	32(16.0%)	157	46(29.0%)	355	78(22.0%)	<0.001
Chickpea(*Cicer arietinum*)	198	40(20.0%)	157	25(16.0%)	355	65(18.0%)	<0.001
Peanut(*Arachis hypogaea*)	198	18(9.0%)	157	35(22.0%)	355	53(15.0%)	<0.05
Pigeon pea(*Cajanus cajan*)	198	30(15.0%)	157	11(7.0%)	355	41(11.5%)	>0.05
Black gram(*Vigna mungo*)	198	11(5.5%)	157	28(18.0%)	355	39(11.0%)	>0.05
Green gram(*Vigna radiata*)	198	28(14.0%)	157	11(7.0%)	355	39(11.0%)	>0.05
Soya bean(*Glycine max*)	198	20(10.0%)	157	14(9.0%)	355	34(9.5%)	>0.05
Pea(*Pisum sativum*)	198	19(9.5%)	157	5(3.0%)	355	24(6.7%)	>0.05
Lentil(*Lens esculentus*)	198	1(0.5%)	157	6(4.0%)	355	7(2.0%)	>0.05
Cowpea(*Vigna sinensis*)	198	0	157	6(4.0%)	355	6(2.0%)	>0.05

*p-values were calculated using Fisher's exact test.

### Patients' sera

Blood was collected from 208 patients with a history of food allergy and SPT positivity to the respective legume(s). Blood was also collected from 20 healthy non-allergic individuals (controls) with negative skin reactivity to different allergen extracts. Cases and controls were both sex and age matched.

### Estimation of Specific IgE

The levels of sIgE in SPT positive patients' sera was determined by ELISA following the protocol of Singh *et al.* [25]

### Estimation of Total serum IgE

Total serum IgE was estimated in sera of patients using kit procured from Bethyl Laboratories (USA) as described earlier [26]. IgE values (1 IU/ml = 2.4 ng/ml) were calculated using the standard curve.

### Stripped Basophil histamine release assay

Histamine release assay was performed in 30 kidney bean sensitive individuals having significantly high sIgE values following the protocol by Kukreja *et al* [27]. In brief peripheral blood was drawn from nonallergic donors and mixed (1 : 5 v/v) with 6% dextran in saline containing 0.01 M EDTA and 2% dextrose. After 90 min, the leukocyte-rich upper layer was drawn, centrifuged and washed twice with saline. The basophils in suspension were stripped off bound IgE by incubation with lactic acid buffer for 3.5 min [28](Kleine Budde et al., 2001). Following incubation, cells were washed in HEPES buffer. Subsequently, the cells were resensitized with a sensitization mixture that contained sera with elevated IgE against kidney bean allergen (n = 30). Cells sensitized with nonallergic sera (n = 5) served as control. The histamine release assay was standardized using a graded amount of protein (1 ng–1 mg), and the protein concentration inducing optimal histamine release (5 ng) was selected for the assay. After passive sensitization, cells were stimulated with kidney bean protein (5 ng) in HEPES buffer containing 1 mM CaCl_2_. After a 1-h incubation, the reaction was stopped by the addition of icecold 0.9% NaCl (w/v). After centrifugation, the cell-free supernatant containing histamine was taken in a fresh Eppendorf tube and mixed with 12% perchloric acid (Sigma). The histamine content was determined by the fluorometric method, using o-phthalaldehyde (Sigma). Spontaneous histamine release was measured in the supernatant of unstimulated cells. The total histamine content was determined by lysis of cells with 3% perchloric acid. The allergen-induced histamine release was calculated as a percent of the total histamine content after correcting for spontaneous release.

### Sodium dodecyl sulphate-polyacrylamide gel electrophoresis (SDS-PAGE) and Immunoblot

To study the protein profile, raw and boiled kidney bean extracts (20 µg protein per lane) were loaded on a 12% reducing gel [29]. Protein profile of boiled (15, 30, 45 and 60 min at 100°C) kidney bean was also analysed by SDS-PAGE. The resolved proteins were stained with Coomassie brilliant blue R-250 (CBB).

For immunoblot, the resolved proteins were transferred on to nitrocellulose membrane as described by Towbin *et al.* [30]. The unbound sites were blocked by 3% bovine serum albumin for 3 h at 37°C. The NCM strips were washed and incubated with 1∶10 v/v kidney bean-hypersensitive patients' sera at 4°C. Healthy serum pool was taken as control. Kidney bean positive patients' serum pool was also incubated with *Curvularia lunata* (unrelated) extract to check the nonspecific binding. The strips were washed with PBS–Tween 20 and incubated with 1∶1000 diluted antihuman IgE-peroxidase (Sigma, USA). The IgE binding was detected by diaminobenzidine with hydrogen peroxide in sodium acetate buffer (pH 5.0). Immunoblotting was also performed with other legume extracts using kidney bean positive patients' sera.

### Excision of bands and In-gel digestion of kidney bean protein

Protein bands corresponding to 8 major allergens identified by immunoblot were excised from coomassie stained gel and subjected to trypsin digestion described by O'Cualain *et al*. [31] with slight modification.

### Mass spectrometric analysis

Peptide solution was injected for analysis by nLC-MS/MS (Agilent, Palo Alto, CA, USA) using Agilent 1100 NanoLC-1100 system following manufacturer's instructions. In brief, the samples (6 µL) were concentrated on pre-column (Zorbax 300SB-C18, 150 mm 675 mm, 3.5 mm) and after 5 min, the pre-column was connected with the separating column, and multistep gradient was started. An LC/MSD Trap XCT with a nano-electrospray interface operated in the positive ion mode was used for MS. Ionization was performed with a liquid junction and a noncoated capillary probe. Peptide ions were analyzed by the data-dependent method as follows: full MS scan. The scan sequence consists of 1 full MS scan followed by 4 MS/MS scans of the most abundant ions. Data were analyzed using Agilent Ion trap Analysis software version 5.2 and proteins were identified by database search against the MASCOT database.

### ELISA inhibition

The allergenic potency of kidney bean extract and its cross reactivity with other legumes was determined by ELISA inhibition. Inhibition of IgE binding was assessed with serum pools of kidney bean positive patients' preincubated with 5, 10, 50, 100, 500, 1000 and 10000 ng of legumes namely kidney bean, black gram, chickpea, lentil, pea, peanut and pigeon pea as inhibitors. The mixture was added to the solid phase bound raw kidney bean extract in ELISA plate. Here kidney bean positive patients' pooled sera without inhibitor was taken as a positive control. To check cross-reactive carbohydrate determinants specific inhibition, if any, kidney bean positive patients' pooled sera was preabsorbed with bromelain (Sigma) and used for ELISA. ELISA inhibition was also carried out using *Curvularia lunata* extract as an inhibitor. Percentage inhibition was calculated as described below.

1- OD of sample with inhibitor X 100

OD of sample without inhibitor

### Immunoblot inhibition

Immunoblot inhibition was performed to establish cross-reactivity of kidney bean with six common legumes. Only kidney bean positive sera from 7 patients' (> 1.0 O.D.) were pooled and preincubated with 500 µg of kidney bean (homologous extract) and legume extracts namely black gram, chickpea, lentil, pea, peanut and pigeon pea, separately. Inhibition was also performed with *Curvularia lunata* extract and bromelain as inhibitors. The proteins were transferred on to nitrocellulose membrane, strips were cut, blocked and incubated with the preincubated pooled sera, separately. The rest of the procedure was similar to immunoblotting.

### Statistical analysis

Values are represented as mean±SD. Correlation analysis was carried out to study the association among SPT, sIgE, total IgE and basophil histamine release using MS EXCEL, Prism V software (Graph Pad Prism, San Diego, California, USA). Epi Info 3.3.2. was used to calculate the significance of SPT positivity among the selected 10 legumes. The significance level was considered to be p<0.05 (two-tailed).

## Results

### Sensitization to legume extracts

The prevalence of sensitization (SPT +ve) to legumes was observed in 208 (58.0%) patients and it varied from 101 (49.0%) at Bangalore to 107 (68.0%) at Chandigarh. Sensitization (SPT +ve) to kidney bean was in maximum 78 (22.0%; p<0.001) patients followed by chickpea 65 (18.0%; p<0.001), peanut 53 (15.0%; p<0.05) and to other legumes in <12.0% (p>0.05) cases (Table 2). Besides legumes, some of the patients were also SPT +ve to aeroallergens which may be due to co-sensitization or cross reactivity.

Total IgE (88–2175 IU/ml) and sIgE (0.178–3.166) were elevated in SPT +ve legume cases indicating sensitization. sIgE estimation showed sensitization to kidney bean in maximum 70 (89.7%) patients followed by lentil 3 (42.9%), soybean 14 (41.2%), pigeon pea 16 (39.0%) and black gram 15 (38.4%) (Table 3). The cut off value to define ELISA positive was 0.138 (OD_492_) i.e. ≥ three times of the normal control. Intensity of SPT reaction has been found to be very well correlated with sIgE (r = 0.85, p<0.0001) but not with the total IgE levels (r =  −0.16, p = 0.3522).

**Table 3:** Sensitization to legume allergens detected by sIgE estimation in ELISA.

Legume extract	SPT patients positive	ELISA positive cases*(% sensitization)	sIgE (OD_490_)
Kidney bean	78	70 (89.7)	0.196–3.166
Chickpea	65	7 (10.8)	0.192–0.693
Peanut	53	14 (26.4)	0.182–1.102
Pigeon pea	41	16 (39.0)	0.199–0.711
Black gram	39	15 (38.4)	0.178–1.035
Green gram	39	3(7.7)	0.215–0.330
Soybean	34	14 (41.2)	0.198–0.637
Pea	24	7 (29.2)	0.187–0.471
Lentil	7	3 (42.9)	0.216–0.395
Cow pea	6	2(33.3)	0.198–0.575

*Cut off value for sIgE positivity = 0.138 OD (≥3 times of control).

Interestingly, during the follow up study, the patients (history, SPT and sIgE positive) showed reversal of symptoms (back to normal) once the offending food was withdrawn from their diet.

### Stripped basophil histamine release assay

**Figure 1:**
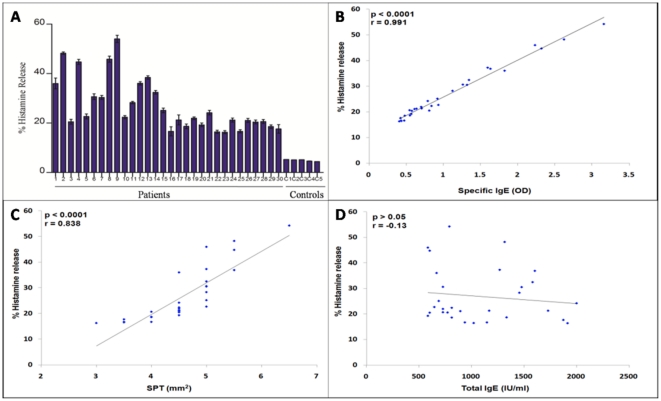
Histamine release assay. Basophils in leukocyte suspension were stripped off the bound IgE. Stripped basophils were sensitized with sera of kidney bean hypersensitive patients. The sensitized basophils were stimulated with kidney bean extract. The Histamine released from 30 patients varied from 16% to 54% while in controls it is less than 6% (A). Scatter plot of the correlation analysis between % histamine release vs SPT (mm^2^) (B), % histamine release vs sIgE (C) and % histamine release vs total IgE (D) among 30 kidney bean sensitive individuals. Basophil histamine release has been found to be very well correlated with SPT (r = 0.83, p<0.0001) and sIgE (r = 0.99, p<0.0001) but not with total IgE (r =  −0.13, p = 0.4942).

Basophils sensitized with kidney bean sensitive individual patients' sera (IgE) exhibited histamine release on challenge with kidney bean extract in the range of 16–54% (Table 4, Figure 1A). Basophils sensitized with control sera did not show more than 5% histamine release. Histamine release was found to be significantly correlated with SPT (r = 0.83, p<0.0001) (Figure 1B) and sIgE (r = 0.99, p<0.0001) (Figure 1C) but did not correlated statistically with total serum IgE levels (r =  −0.13, p = 0.4942) (Figure 1D) among 30 individuals.

**Table 4:** Estimation of sIgE, total IgE and basophil histamine release among the 30 kidney bean sensitive (SPT +ve) patients and 5 controls (SPT -ve).

Patient No.	Age/Sex	Wheal size of histamine	Wheal size ofKidney bean extract	SIgE (O.D.)	Total IgE (IU/ml)	Histamine Release (%)
1	51/F	7x7	6x3	1.822	0667	35.99±3.76
2	34/F	5x5	6x5	2.621	1317	48.22±0.86
3	32/M	7x5	5x4	0.808	0771	20.54±1.71
4	35/M	5x6	6x5	2.318	0604	44.75±1.73
5	59/M	7x5	5x5	0.932	0646	22.67±1.76
6	34/M	7x4	5x5	1.262	0729	30.66±2.00
7	40/F	7x6	5x5	1.316	1479	30.49±1.41
8	52/M	6x6	6x4	2.230	0583	46.00±2.12
9	40/F	6x5	7x6	3.161	0791	54.24±2.50
10	25/F	7x5	5x4	0.839	0812	22.37±1.13
11	60/M	7x5	5x5	1.121	1458	28.25±0.84
12	32/M	6x6	5x6	1.631	1604	36.06±1.17
13	28/M	5x6	5x5	1.593	1271	38.40±1.25
14	24/M	7x6	5x5	1.342	1583	32.42±1.29
15	32/M	6x5	5x5	0.919	0688	25.11±1.63
16	48/M	6x5	4x4	0.471	1146	16.63±3.17
17	23/F	7x5	5x4	0.704	1167	21.27±3.49
18	57/F	7x6	4x4	0.547	1333	18.66±1.69
19	22/F	6x5	5x4	0.700	0729	21.96±0.92
20	28/F	7x5	4x5	0.565	0583	19.27±1.29
21	63/F	5x5	5x4	0.789	2000	24.20±1.62
22	49/M	7x5	4x3	0.434	1020	16.42±1.10
23	48/M	5x5	3x3	0.407	1916	16.30±1.09
24	62/F	8x4	5x4	0.636	1729	21.22±1.42
25	56/F	7x4	4x3	0.426	0938	16.65±1.11
26	24/M	7x6	4x5	0.610	0896	21.09±1.41
27	8/M	6x6	4x5	0.576	0604	20.45±1.37
28	23/F	6x5	4x4	0.546	0729	20.62±1.38
29	52/M	7x5	4x4	0.479	0813	18.56±1.24
30	35/F	7x5	3x4	0.422	1875	17.62±3.00
C1	28/M	5x4	1x1	0.047	57	5.27±0.32
C2	32/M	4x5	1x2	0.105	52	5.11±0.41
C3	26/F	5x4	1x1	0.870	129	5.16±0.98
C4	22/F	4x3	2x1	0.029	77	4.64±0.67
C5	38/M	3x3	1x1	0.047	48	4.40±0.25

### Protein profile of kidney bean extract

**Figure 2:**
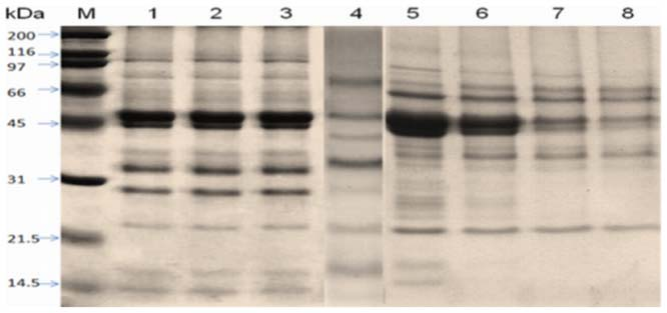
SDS-PAGE – profile of three batches of raw kidney bean extract Kb1, Kb2 and Kb3 (lane 1, 2, 3 respectively), kidney bean boiled for 15 min at 121°C (lane 4) and boiled at 100°C for 15, 30, 45 and 60 min (lanes 5–8) stained with Coomassie brilliant blue. M: molecular weight markers.

Kidney bean contained 71, 62, and 64 mg protein/g of dry powder in three batches of extracts. SDS-PAGE resolved raw kidney bean extract into 22 coomassie stained protein bands of 14 to 150 kDa (Figure 2, lane 1–3). The extract from boiled kidney bean for 15, 30, 45 and 60 min at 100°C resolved into 19,14,9 and 7 bands whereas the extract from boiled kidney bean (15 min at 121°C) separated into 13 bands on SDS-PAGE (Figure 2, lane 4–8). A new band appeared at 40 kDa in the extract boiled for 15 min at 100°C (Figure 2, lane 4).

### Allergenic proteins of kidney bean extract

**Figure 3:**
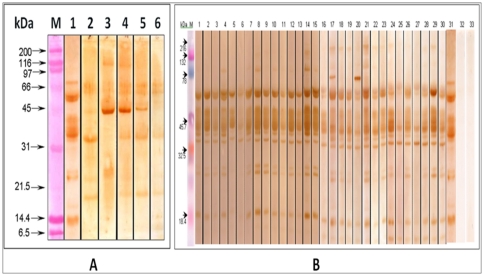
Immunoblot of raw kidney bean (lane 1), kidney bean boiled for 15 min at 121°C (lane 2) and boiled at 100°C for 15, 30, 45 and 60 min (lanes 3–6) probed with pooled patients' sera (1∶10 v/v). M: molecular weight markers (A). Immunoblot of kidney bean probed with individual patients' sera. (Lanes 1–30), pooled patients' sera (Lane 31), pool of normal human sera (Lane 32), *Curvularia lunata* extract tested with kidney bean sensitive patients' pooled sera (Lane 33), M: Molecular weight maker (B).

The IgE binding components of extracts prepared from raw and boiled kidney bean were analyzed by western blot with pooled sera of kidney bean-sensitive patients (n = 30) showing SPT reactivity more than 2+ and significant raised sIgE (Table 4, Figure 3A). Raw kidney bean extract showed 15 IgE-binding proteins of 120, 95, 70, 58, 55, 50, 45, 42, 40, 37, 34, 26, 24, 18 and 16 kDa (Figure 3A, lane 1) whereas boiled kidney bean for 15 min at 121°C showed only 5 IgE binding protein bands of 58, 55, 37, 24 and 18 kDa (Figure 3A, lane 2). Boiled kidney bean for 15, 30, 45 and 60 at 100°C showed 7, 6, 5, and 3 allergenic protein bands, respectively (Figure 3A, lane 3–6). The extract showed heterogeneity in the banding pattern with eight major allergens of 58, 50, 45, 42, 40, 37, 34 and 18 kDa recognized by ≥95.0% of the patient's sera. The proteins of 26 and 24 kDa were recognized by 77.0% of patients' sera. The strips incubated with normal healthy sera did not show any binding. Kidney bean-positive patients' sera incubated with *Curvularia lunata* (unrelated) extract could not recognize any protein (Figure 3B).

### Mass spectrometric analysis

Analysis of 8 major allergens (protein bands) of kidney bean (Figure 3B) and detailed characterization of identified protein (Table 5) by nLC-MS/MS resulted in identification of 4 proteins, assigned as Alpha-amylase inhibitor precursor from *Phaseolus coccineus*, Full  =  erythroagglutinating phytohemagglutinin; AltName: Full = PHA-E, phaseolin from *Phaseolus vulgaris*, and group 3 late embryogenesis abundant protein from *Phaseolus vulgaris*.

**Table 5:** Peptide mass fingerprint database search of selected spots from 1-DE of kidney bean proteins.

Spot No.	Accession no.	Identification	Matches (Ion score >50)^a^	Sequence Coverage^b^ (%)	MASCOT score^c^	Tht mass/pI (kDa)	Obs mass (kDa)	Proposed function
1	CAH60259	Alpha-amylase inhibitor precursor [Phaseolus coccineus]	17(9)	22	256	27.0/5.17	18	defensive protein
2	P05088	Full = Erythroagglutinating phytohemagglutinin; AltName: Full = PHA-E	39(21)	32	593	29.7/5.15	34	defensive protein
3	CAD29133	lectin [Phaseolus vulgaris]	19(7)	21	278	29.6/4.83	37	defensive protein
4	CAD29133	Lectin [Phaseolus vulgaris].	54(32)	26	494	29.6/4.83	40	defensive protein
5	AAA99534	Phaseolin [Phaseolus vulgaris]	32(19)	32	931	47.5/5.42	42	storage protein
6	P07219	Full = Phaseolin, alpha-type	34(17)	26	766	49.2/5.25	45	storage protein
7	ABA26579	Group 3 late embryogenesis abundant protein [Phaseolus vulgaris]	7(1)	16	328	50.7/5.80	50	Stress tolerant protein
8	ABA26579	Group 3 late embryogenesis abundant protein [Phaseolus vulgaris]	4(2)	6	130	50.7/5.80	58	Stress tolerant protein

**Footnotes:** Abbreviations; Tht  =  Theoretical, Obs  =  Observed. ^a^Matches a  =  Number of peptides matched with protein in MS/MS query. ^b^Sequence coverage b  =  Total percentage of proteins amino acid sequence covered by peptides in MS/MS analysis. ^c^MASCOT score c =  >40 indicate identification or extensive homology (p<0.05).

### Allergenic potency of kidney bean and cross-reactivity with legumes

**Figure 4:**
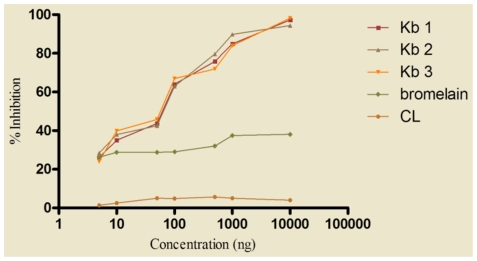
IgE ELISA inhibition of kidney bean extract using self protein as inhibitor. Kidney bean positive patients' pooled sera (1∶10 v/v) was preincubated with 5, 10, 50, 100, 1000, 10000 ng of three batches of kidney bean extract as inhibitors (Kb1, Kb2, Kb3), bromelain and CL (*Curvularia lunata*) were also used as inhibitor for IgE inhibition to kidney bean. ELISA was carried out on solid phase coated kidney bean (2 µg/100 µl/well) and preincubated sera.

ELISA inhibition was carried out to determine the potency of three different batches of kidney bean with similar SDS-PAGE profile. The kidney bean extract was highly potent since it required only 67.3±2.51 ng of homologous (self) protein to achieve 50.0% inhibition of IgE binding in ELISA (Figure 4).

**Figure 5:**
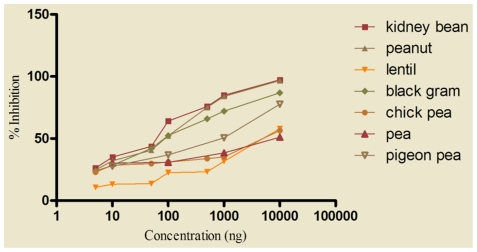
IgE ELISA inhibition of kidney bean extract with self and heterologous legume extracts. For determination of cross reactive proteins between kidney bean and different legume extracts, 7 separate serum pools were prepared with sera of patients' skin test positive to kidney bean but negative to that of legume extract to be tested. Kidney bean positive patients' pooled sera (1∶10 v/v) was preincubated with 5, 10, 50, 100, 1000, 10000 ng of legume extracts namely black gram, chickpea, kidney bean, lentil, pea, peanut, soybean and pigeon pea as inhibitors. ELISA was carried out on solid phase coated kidney bean (2 µg/100 µl/well) and preincubated sera.

To assess cross reactivity of kidney bean with 6 different legumes namely black gram, chickpea, lentil, pea, peanut and pigeon pea, ELISA inhibition was performed using different serum pools and respective legume extract as inhibitor. Sixty seven ng of kidney bean protein (homologous) was required to obtain 50.0% inhibition of IgE binding in ELISA, whereas both peanut and black gram (hetrologous) caused same inhibition to solid phase kidney bean with 85 ng of protein(s) showing extensive cross reactivity (Figure 5). The extracts of pigeon pea, chickpea, lentil and pea produced 50.0% inhibition with 1000, 7500, 7500 and 10000 ng of protein(s), respectively. Preabsorption of pooled patients' sera with even 10 µg of *Curvularia lunata* extract resulted in only 8% inhibition to solid phase kidney bean extract. However, bromelain inhibited 38.0% of IgE binding in ELISA at a concentration of 10 µg (Figure 4).

**Figure 6:**
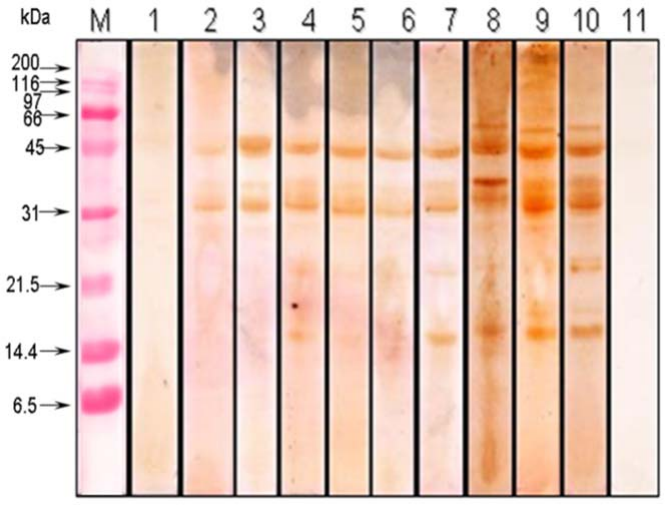
Immunoblot inhibition of kidney bean extract with self and other legume extracts. Kidney bean positive pooled patients' sera was preincubated with 500 µg of extracts namely kidney bean (lane 1), peanut (lane 2), black gram (lane 3), chickpea (lane 4), lentil (lane 5), pigeon pea (lane 6) and pea (lane 7). Bromelain (lane 8) and *Curvularia lunata* (lane 9) were also used as inhibitor to assess non specific binding. Immunoblot using kidney bean positive pooled patients' sera without inhibitor was used as positive control (lane 10) and normal human sera (lane 11) as negative control.

To establish the cross reactivity of kidney bean allergens immunoblot inhibition was performed using kidney bean positive patients' pooled sera and self/respective legume extract as inhibitor. Electrophoretically transferred kidney bean proteins were incubated with preinhibited serum pool. Preabsorption of pooled patients' sera with 500 µg of kidney bean extract (self) totally abolished its IgE reactivity (Figure 6, lane 1). For cross inhibition, maximum bands of kidney bean protein were inhibited by peanut followed by pigeon pea, black gram, lentil, chickpea and pea used as inhibitor (Figure 6, lane 2–7). Peanut, pigeon pea and black gram could inhibit IgE binding of 120, 95, 70, 40, 37, 26, 24, 18, and 16 kDa proteins in kidney bean indicating presence of cross reactive components. Reduction in IgE binding of high-molecular-weight kidney bean proteins (>70 kDa) was also recorded on preabsorption of sera with each legume extract(s) tested. However, IgE binding of 55, 50, 45 and 42 kDa proteins could not be inhibited by other legume extract(s). Therefore it can be inferred that these proteins are specific allergens of kidney bean. Preabsorbed sera with bromelain and *Curvularia lunata* did not show any inhibition (Figure 6, lane 8, 9).

## Discussion

Studies have suggested that patient's history, skin testing, provocation tests and *in vitro* assays provide information on sensitization to offending food(s) [14], [17], [32]. The present study was undertaken to identify sensitization to predominant legumes in a group of allergic rhinitis and asthma patients (physician diagnosed) from India. In addition, IgE binding components of kidney bean and its cross reactivity among 6 common legumes was also investigated.

Orange *et al.* [33] reported 64.0% prevalence of food allergy in children with atopic dermatitis and asthma or other respiratory diseases. Ibanez *et al.* [34] found legumes as the 5^th^ most prevalent source of food allergy with chickpea and lentil as predominant cause of allergic reaction in Spanish children. Shaikh [35] observed sensitization to legumes in 20.0% patients of Mumbai, India. A study by Gupta *et al.* [36] in Eastern India accounts 51.0% of adult food allergy with legumes. In the present study, 58.5% of patients showed sensitization to one or more legume(s) based on history and SPT results. Kidney bean demonstrated sensitization (SPT +ve) in maximum patients 78 (22.0%) followed by chick pea 65 (18.0%), peanut 53 (15.0%), pigeon pea and black gram (11% each). Previously, Kumari *et al.* [14] observed sensitization to black gram in only 1.7% of patients. Similar to Misra *et al.* [37], chickpea (15.5%) was a potent sensitizer in the present study also (18.0%). sIgE was also found elevated significantly in majority of legume sensitive cases. Seventy out of seventy eight SPT positive patients to kidney bean have raised sIgE (Table 3). Patients showed signs of recovery from the symptoms once they avoided the suspected food, this further confirmed sensitization in them.

Earlier, the biopotency of few allergenic molds have been determined by histamine release assay [38], [39]. Its utility has also been depicted in the diagnosis of hen's egg, cow's milk, and wheat allergy with an efficiency of more than 70% [40]. In the present study, kidney bean induced significant histamine release from basophils sensitized with kidney bean positive patients' sera which correlated significantly (p<0.05) with SPT and sIgE. This suggests the importance of the combination of SPT, sIgE and histamine release in the diagnosis of food allergy, as these three parameters are found to be significantly correlated with each other.

Immunoblotting with hypersensitive patients' sera has been used successfully to identify IgE binding components of allergen extracts [14], [17], [37]. Pascual *et al.* [16] showed 16 IgE-binding proteins in lentil, of which 54 and 38 kDa proteins were important allergens while Ibanez *et al.* [17] showed six allergenic proteins between 69 and 18 kDa recognized by more than 50.0% of patients, and a 53 kDa protein by 92.0% of the patients. Patil *et al.* [13] reported five major allergens between 70 and 20 kDa in chickpea. Kumari *et al.* [14] identified 8 major IgE binding components of 78–16 kDa in black gram. In the present study, immunobloting demonstrated eight major allergens of 58, 50, 45, 42, 40, 37, 34, 18 kDa in raw kidney bean extract detected by ≥95.0% of patients' sera. Boiling resulted in the loss of high molecular weight (>70 kDa) IgE binding proteins.

The 8 major allergens of kidney bean identified by immunoblotting were subjected to nLC-MS/MS analysis. Of these, 4 showed significant matches to known proteins in database. Two proteins were identified as lectin and alpha-amylase inhibitor, these are known to provide defence against insects, [41] one as phaseolin, the main reserve globulin in kidney bean and the fourth belongs to late embryogenesis abundant (LEA) proteins with a role in protecting other proteins from aggregation due to desiccation or osmotic stresses associated with low temperature [42]. Rouge *et al.* [19] also identified phaseolin and PHA as putative allergens in kidney bean. Both phaseolin (>150 kDa) and PHA (120 kDa) consist of oligomeric proteins resistant to heat denaturation and digestive proteolysis, exhibiting an extended surface susceptible to display IgE-binding epitopes that probably account for their allergenic propensity. Phaseolin and PHA are closely related to the Ara h 1 vicilin and the Ara h agglutinin (PNA) allergens from peanut. In addition to the cupin allergens, lectins appear to be potentially allergenic proteins of edible legumes [19].

Cross reactivity has been reported previously among some legume allergens by ELISA inhibition [14], [33]. In the present study, kidney bean required 67.3 ng of homologous protein for 50.0% inhibition of IgE binding in ELISA whereas black gram and peanut produced same inhibition with 85 ng of protein showing extensive cross reactivity. Other legume extracts such as pigeon pea caused 50.0% inhibition with 1000 ng protein whereas both chickpea and lentil required 7500 ng of protein for the same. Further, IgE binding protein components of kidney bean were completely inhibited when pooled patients' sera was preabsorbed with 500 ng of self protein in immunoblot inhibition. Peanut, pigeon pea and black gram could inhibit protein bands of 120, 95, 70, 40, 37, 26, 24, 18, and 16 kDa in kidney bean suggesting presence of shared allergens.

In conclusion, kidney bean as well as chickpea, peanut, cowpea, lentil and soybean are recognized as important sensitizers by SPT and ELISA. In addition, we have identified 8 major IgE binding components from kidney bean and demonstrated its cross reactivity with peanut, black gram and pigeon pea. Although DBPCFC test is considered to be the gold standard in the diagnosis of food allergy but it presents practical as well as ethical problems and suffers from several pitfalls. Further, DBPCFC is difficult to perform in normal outpatient clinics, therefore, this procedure is rarely performed outside the academic context [43]. We demonstrated three parameters SPT, sIgE and basophil histamine release to be significantly correlated with each other and therefore important tool in the diagnosis of food sensitization.
